# Commonalities in Management and Husbandry Factors Important for Health and Welfare of Captive Elephants in North America and Thailand

**DOI:** 10.3390/ani10040737

**Published:** 2020-04-23

**Authors:** Janine L. Brown, Pakkanut Bansiddhi, Jaruwan Khonmee, Chatchote Thitaram

**Affiliations:** 1 Center for Species Survival, Smithsonian Conservation Biology Institute, Front Royal, VA 22630, USA; 2 Center of Elephant and Wildlife Research, Faculty of Veterinary Medicine, Chiang Mai University, Chiang Mai 50100, Thailand; pakkanut.vet@gmail.com (P.B.); jaruwan.khonmee@cmu.ac.th (J.K.); 3 Department of Companion Animals and Wildlife Clinics, Faculty of Veterinary Medicine, Chiang Mai University, Chiang Mai 50100, Thailand; 4 Department of Veterinary Bioscience and Veterinary Public Health, Faculty of Veterinary Medicine, Chiang Mai University, Chiang Mai 50100, Thailand

**Keywords:** Asian elephant, health, welfare, body condition, glucocorticoids, zoos, tourist camps, Thailand, insulin, glucose, lipids, North America

## Abstract

**Simple Summary:**

There is considerable concern about the welfare of elephants used for education, research, and entertainment purposes in western zoos and Asian tourist camps, and whether captive venues meet the needs of these highly social and intelligent animals. Therefore, it is important to conduct studies to determine how factors in the captive environment affect animal welfare both positively and negatively. The use of multi-disciplinary approaches to assess welfare has aided improvements in the management of captive elephants in North American zoos and Thailand tourist camps. In this paper, we review the most recent findings of our elephant studies that, using similar methodologies, highlight the importance of proper diets, adequate exercise, natural and stimulating environments, and freedom of movement to welfare. Overall, we found a number of commonalities in how environmental factors affect biological function of elephants managed under vastly different conditions. This type of integrative information is important for establishing welfare guidelines to ensure healthy, sustainable global populations of captive elephants.

**Abstract:**

This review paper is a synthesis of results from multiple studies that we have conducted over the past several years using similar methodologies to identify factors related to welfare of captive populations of elephants in North American zoos and Thailand tourist camps. Using multiple conservation physiology tools, we found that, despite vastly disparate management systems, there are commonalities in how environmental and husbandry factors affect physical and physiological outcomes. Elephants appear to have better welfare, based on fecal glucocorticoid metabolite (FGM) analyses, when housed under conditions that provide a more enriched, stimulating, and less restrictive environment. We also found it is essential to balance diet and exercise for good body condition and metabolic function. In Thailand, use of tools to control elephants, such as the ankus (i.e., guide, hook) and chains, did not equate to poor welfare per se, nor did riding; however, improper uses were associated with higher wound scores and FGM concentrations. Foot health was good overall in both regions, with cracks being the most common problem, and better foot scores were found in elephants kept on softer substrates. Based on these findings, science-based guidelines are being developed in Thailand, while in North America, changes are being incorporated into elephant standards and husbandry resource guides. Management across venues can be improved by encouraging elephant exploration and exercise, establishing socially compatibility groups, ensuring proper use of tools, and providing balanced diets. We contend there is no “one-size-fits-all” management strategy to guarantee good welfare for elephants, but there are essential needs that must be met regardless of where or how they are managed. Future studies are needed to find ways to better socialize elephants; determine how temperament affects coping styles and resilience; study the importance of good handler-elephant relationships; identify more ways for elephants to engage with the environment; and assess the effect of life history on subsequent physiological and psychological well-being.

## 1. Introduction 

Elephants are vital keystone, umbrella, and flagship species and have significant impacts on ecosystems and the species therein, in addition to raising awareness for conservation action [1,2]. Despite their importance, however, wild Asian elephants are endangered because of human–elephant conflict, habitat destruction, and poaching for ivory, skin, and other products [3]. About a third of Asian elephants today are managed under human care, used in religious, cultural, educational, and tourist activities [4]. Though not a panacea, captive breeding can aid species conservation efforts [5,6,7], and in the case of elephants, serve as sources of animals for reintroduction [8,9]. Most captive Asian elephant populations are not self-sustaining, however, due to higher mortality than birth rates. This is true for North America [10] and many Asian range countries, including Thailand [11]. In addition, the welfare of captive elephants has come under increased scrutiny, with animal activist groups claiming the physical and psychological needs of elephants cannot be met [12,13]. There are roughly 140 Asian elephants in more than 30 American Zoo and Aquarium (AZA)-accredited zoos in North America, while Thailand has >3500 captive elephants, ~75% of which participate in tourist activities such as shows, riding, feeding, and bathing [14]. Understanding how factors in the captive environment impact health, reproduction, and behaviour is key to optimizing management protocols. To that end, animal welfare science has developed into a field that can guide improvements in husbandry through research that is multi-disciplinary, multi-institutional, and holistic. In most cases, the causes of welfare problems are multifactorial, and it is virtually impossible to study all at once or determine how they interact using traditional experimental approaches. For elephants, these can vary by region (western zoos vs. range country facilities), but it is not unreasonable to assume there are commonalities in what elephants need no matter how or where they are managed. Thus, adoption of more epidemiologically based approaches could contribute to a better understanding of what, out of many possible inputs, are most important for good welfare.

Epidemiology identifies patterns in specified populations to understand the prevalence of hazards and the risk factors associated with their occurrence. Once known, effective strategies can then be developed to minimize those risks [14]. While mostly applied to disease risks, recent integrative studies conducted by our team utilized a variety of tools and analytical techniques to assess how the captive environment affects physiological and psychological wellbeing of Asian elephants in North America [15,16,17,18,19,20,21,22,23,24,25] and Thailand [26,27,28,29,30]. The North American studies involved ~90% of elephants housed in American Zoo and Aquarium (AZA) facilities [31] and was followed a few years later by a series of comparable studies using similar methodologies in Northern Thailand tourist camps. Information on husbandry, management, and facilities was collected, and within each region, the results were correlated to assessments of fecal glucocorticoid metabolite (FGM) concentrations as an indicator of stress, physical health, body condition, serum metabolic markers, lipid profiles, and behavior. The goal of this review paper was not to summarize elephant welfare studies in general but to compare and contrast results of our collective works and highlight the commonalities in husbandry practices and environmental factors related to physical and physiological outcomes in North America and Thailand despite vastly different management. From these studies, we conclude there is no “one-size-fits-all” way to guarantee good welfare. Rather, there are essential physical and psychological needs that must be met regardless of where or how elephants are managed and/or used.

## 2. Methodology

### 2.1. Research Populations

#### 2.1.1. North America

Asian elephants (26 males, 91 females) were enrolled in the Using Science to Understand Zoo Elephant Welfare study conducted between 2012 and 2014 by co-author Brown and more than a dozen collaborators to identify factors in the captive environment that have negative and positive effects on welfare. Data were collected on management and husbandry practices [32], behaviour [15], physiology [16,17,18,19,20,21,22], and health [23,24]. Details on population age structures are provided in those publications. Information was used to explore relationships between factors in the captive environment and welfare outcomes [32] (Table 1).

Body condition was assessed based on three standardized photographs (lateral, rear, and rear angle) of key body regions (ribs, backbone, and pelvic bone) to assign a score on a five-point scale, with 1 representing the lowest and 5 representing the highest body condition score (BCS) [20]. Blood was collected every two weeks for one year from an ear or leg vein, and the serum analyzed for insulin using a solid-phase, two-site bovine insulin enzyme immunoassay (EIA, Mercodia, Uppsala, Sweden) and glucose to evaluate metabolic function [19,20]. Foot health was assessed during a single physical examination conducted at the midpoint of the study. Information recorded included the presence or absence of issues with the pad, sole, or toenail, such as cracks, defects, ulcerations, bruises, fissures, abscesses, or horn growth [23]. An abnormality in any location was assigned a score of 1, such that each foot could have a maximum score of 3, with each elephant having a maximum score of 12. Presence of skin lesions and wounds also were recorded. Fecal samples were collected bi-weekly for one year to assess FGM concentrations as described in Brown et al. [17]. Briefly, frozen samples were lyophilized and 0.1 g (±0.02) of well-mixed fecal powder was extracted twice with 80% methanol using a multi-tube vortexer. Supernatants were combined, dried, reconstituted in 1 mL of 100% methanol, dried again, and then sonicated for 30 s in phosphate buffer. Samples were diluted (1:8) in assay buffer for analysis by a double-antibody EIA using a polyclonal rabbit anti-corticosterone antibody (CJM006). Stereotypic behavior was assessed in subsets of the study population through video data collected during the daytime on 42 elephants (eight male, 34 female) for 1 h per week during winter (December–February) and summer (June–August) [15]. Finally, nighttime behavior was captured in 13 Asians (three male, 10 female) using night vision cameras, 20 min/hr, in the fall (September–November). Study populations varied because not all zoos were able to collect stereotypic and nighttime behavior due to logistical (e.g., lack of video cameras, poor visibility) and/or staff limitations.

#### 2.1.2. Thailand

As data from the North American study were being analyzed and published, a series of studies by the co-authors was conducted between 2015–2019 in Thailand to determine if similar relationships between management and welfare outcomes existed for tourist elephants. The first study involved a survey that provided information on demographic, housing, and management factors of 627 adult elephants (156 male, 471 female) at 33 camps in Northern Thailand [4]. Questionnaire interviews with camp owners, managers, and veterinarians captured data on camp location, numbers of elephants, programs for tourists, work activities, and elephant management (e.g., nutrition, feeding, water, rest area, working, and healthcare). In general, tourists interacted with elephants through riding programs (bareback or with a saddle), walking with elephants, and feeding of supplementary foods. At some venues, elephants were also used in shows, including painting. As in the North American study, a set of independent variables were developed to compare with environmental factors (Table 1).

From that survey, a subset of 122 adult elephants (33 male, 89 female) at 15 elephant camps in the Chiang Mai area were further evaluated to assess factors associated with physical health outcomes [29]. This subset was based on location; i.e., within 60 km of Chiang Mai University to permit multiple veterinary visits over a one-year study. Wound (WS) and foot (FS) scores were created from physical examinations performed approximately every other month by Chiang Mai University elephant veterinarians. Wounds were scored on a three-point scale (0 = none, 1 = minor, 3 = severe) based on Schein et al. [33] and classified as abrasions (the epidermis has been rubbed off); ulcers (a local excavation of the tissue surface that contains inflammatory exudate); abscesses (a collection of pus enclosed in an area of inflamed tissue); lacerations (irregular shaped wound with possible tissue loss); penetrating (a wound caused by a sharp, usually slender object that passes through the skin into the underlying tissues); and incisions (a cut in the skin caused by sharp, cutting materials). The system to assess foot health differed from that of Miller et al. [23] in that each foot was given a score of 0 (no problem), 1 (mild problems), 2 (moderate problems), or 3 (severe problems). The overall score of each elephant was the highest score from all four feet as described by Todd [34]—maximum score = 3, as compared to a maximum of 12 in Miller et al. [23]. The number, location, and direction of nail cracks also were noted. Body condition was scored the same as that for the North American population based on Morfeld et al. [20]. 

In a further subset of elephants (18 males, 66 females at 15 camps) from camps willing to collect fecal samples, FGM concentrations were analyzed in samples collected twice monthly for one year and subjected to multivariable modeling of demographic and management factors [30]. Methods of fecal processing and analysis were similar to those used in North America with three exceptions: (1) wet feces were dried using a conventional oven at 60 °C for ~24–48 h rather than a lyophilizer; (2) crushed feces were not sifted to remove larger fiber particles; and (3) dried feces was extracted with 90% ethanol in a boiling water bath rather than 80% ethanol on a multi-tube vortexer. Mahouts (elephant keepers) were also asked about the presence or absence of stereotypic behaviors. Finally, correlations between management, diet, and work activities, and BCS, FGM, and serum metabolic (insulin, glucose) and lipid markers were examined in a third set of 46 elephants (33 females, 13 males at five camps) [26,27,28] that could be blood sampled. The FGM and insulin EIAs were the same as those employed in the North American studies.

## 3. Research Findings

Descriptive data (mean ± SEM) for the welfare indicators used in the North America and Thailand studies are shown in Table 2, while Table 3 shows factors in the captive environment that correlated with welfare outcomes using Generalized Estimating Equations (R Development Core Team [35]). 

### 3.1. Body Condition and Metabolic Function

Body condition is a simple assessment tool, with scores based on direct observations or photographs. Animals deemed too fat (at the high end of a graded scale, with middle scores indicating a normal condition) can then be targeted for changes in diet and/or exercise to mitigate problems with obesity. High BCS (i.e., excess fat) is a health concern for captive elephants, not only in our two study populations, but also others [36,37] because of potential associations with conditions such as cardiovascular disease, arthritis and foot problems, and ovarian cycle abnormalities [12]. In both North America and Thailand, the majority of elephants were overweight (BCS = 4) or obese (BCS = 5) based on visual assessments. In general, females had higher BCSs than males, and within sex category, elephants in the US had higher scores. The amount of walking-based exercise was a strong predictor of BCS [20,26,27,28], which agrees with other studies showing that walking is an effective means of controlling body fat [38,39,40]. In North America, elephants involved in staff-directed walking of 14 h or more per week had a decreased risk of BCS 4 or 5 [20], whereas in Thailand, better body condition was associated with increased work time and walking distance in elephants used for riding [26,27,28].

A number of feeding variables were related to body condition, such as how and when food was presented, and at least in Thailand, approximately how much. The schedule of feedings was important, with results pointing to implementation of a random rather than predictable feeding schedule being associated with decreased risk of high body condition in zoos. Increased number of feedings of roughage during the day and at night in Thailand also were related to lower BCS. These findings are supported by human studies, in which varying eating times and frequency reduces the risk of cardiovascular disease and obesity [41]. Furthermore, varying the timing of feedings is now seen as an effective method to control weight in people [42]. Eating multiple, small meals at varying times throughout the day rather than a few larger meals at set times may work by suppressing hunger and lowering serum insulin concentrations [43], whereas a low number of predictable meals may increase insulin compared to high frequency, random feedings [44]. Insulin inhibits lipase enzyme activity and increases fat deposition, thus resulting in excess body fat deposition under conditions of high insulin production [45]. Morfeld and Brown [19] found elevated serum insulin concentrations in elephants with a high BCS, so a similar mechanism may contribute to excessive fat deposition in elephants. 

In the North America study, the hypothesis was that a higher Feed Diversity would predict lower BCS because elephants would increase activity as they searched for food, but instead the relationship was positive [20]. One possibility is that when greater numbers of different feeding methods are used more frequently, there may be an increase in the quantity of food provided/consumed as well; thus, careful monitoring of intake is important if high BCS is a problem. Given that only 20% of zoos utilized unpredictable feeding schedules, and the majority of elephants participated in less than 1 h of walking-based exercise per week at the time of the welfare assessment [46], there is significant potential for addressing poor body condition by applying the management changes indicated by these analyses. Indeed, several zoos have since installed automatic feeders and other food delivery devices to encourage exploration and foraging within the exhibits, although we have noted that some elephants develop anticipatory behaviors in response to the installation of automatic feeders, so that needs further study (JLB, Jilian Fazio, personal information). 

In Thailand, there is little diversity in how tourist camp elephants are fed; they are provided fodder throughout the day, even during trekking. However, increased exercise during tourist activities likely helps them maintain better body condition, which was lower on average than in North America. Similarly, those that worked more hours per day in the form of saddle or bareback riding had lower BCSs [26,27,28]. Our recent survey of tourist camps in Thailand noted a shift in how elephants are managed, with newer camps offering more intimate, passive experiences, and less reliance on activities such as riding [4]. However, we propose that if elephants are not used for riding or shows, the amount and caloric content of food must also be reduced. Indeed, a popular activity for Thailand tourists is feeding elephants, particularly bananas and sugar cane, which possess high concentrations of sucrose and other soluble sugars that appear to be contributing to high BCS and metabolic derangements [26,27,28]. Interestingly, a training variable (positive reward/stimuli) in the univariate analysis in North America was associated with high BCS, which suggested the more frequent use of treats during training may contribute to weight problems. Given all that, we advocate for using lower calorie treats, foods with a lower glycemic index, or non-food rewards for training or in association with tourist feeding. 

Being overweight can have physiological consequences. Obesity is the most common component of metabolic syndrome (i.e., hypertension, dyslipidemia, insulin resistance, glucose intolerance) in humans [47,48,49,50] and also occurs in horses [51]—a model species often used to assess nutritional needs of elephants. Both horses and elephants are hind gut fermenters with similar dietary requirements and are both able to utilize large quantities of low-quality, high-fiber foods [52]. Many components of metabolic syndrome are related to high BCS in elephants [19,26,27]. Because elephants in our studies were not fasted before blood sample collection, glucose:insulin (G:I) ratios were calculated, a technique used to detect insulin sensitivity in women [53], with lower values reflecting metabolic derangements. Very overweight elephants in Thailand (BCS = 4.5–5) had higher measures of insulin; total cholesterol; high density lipoproteins (HDL); low density lipoproteins (LDL), glucose, and fructosamine; and a lower G:I, while in North America, G:I was predictive of body condition, with higher BCS being associated with lower G:I. Comparatively, total cholesterol, triglycerides (except US males), glucose and insulin were similar between regions; however, the overall G:I average value for tourist elephants (G:I = 165) was better than that for zoo elephants (G:I = 110), although the ranges overlapped (23–483 versus 14–430, respectively) [27,28].

In both North America [19] and Thailand [26,27], factors important for normal metabolic function mirrored those associated with good body condition, particularly exercise and feeding practices. We contend that elephants may experience metabolic and health problems associated with obesity, similar to other species, which should be a concern for elephant managers, given how many are scored as overweight or obese. Just as with people, good diets and exercise are key components to a healthy life for elephants, no matter how they are used or where they reside.

### 3.2. Physical Health

#### 3.2.1. Foot Scores

Good foot and joint health are critical to the welfare of elephants, with problems being a major reason for euthanasia in western zoos. In 1994, Mikota, et al. [54] published an extensive review of elephant medical records from 69 North American zoos over the course of 84 years and reported that ~50% experienced foot pathologies, while 64% had musculoskeletal problems. Over a decade later, zoos reported foot abnormalities (33%), arthritis (36%), and lameness (18%) continue as problems in their elephants [55], although perhaps not to the same extent. In the most recent North American study, 67.4% were diagnosed with at least one foot problem, although of those, only 8% were major (score = 5 or more out of 12) [23]; 92.4% were associated with the nails, 13.1% with the pads, and 22.8% within the interdigital space. Findings in Thailand were comparable −36% had no foot problems, and 44% had only mild issues, primarily as uncomplicated nail cracks; only 2% of the foot problems were severe [29]. An earlier study of 74 elephants in three camps in Chiang Mai found the majority of elephants (64%) had mild and 3% had severe foot problems [34], while between 2005–2008 the Thai Elephant Conservation Center’s mobile clinic evaluated 1368 elephants of which 136 (9.8%) presented with musculoskeletal problems (lameness, bone fracture) and 44 (3.1%) had foot problems (cracked nail, nail overgrowth, foot-pad cracking) [56].

In 2001, Fowler [57] proposed that lack of exercise, limited space, standing on hard substrates, wet surfaces, and obesity all contribute to poor foot health. In particular, standing or walking on hard substrates can lead to trauma of foot pads, toenails, joints, and other musculoskeletal structures, resulting in cracks, abscesses, bruises, strains, and degenerative joint disease [57,58]. Indeed, multivariable regression modeling of the North American population provided evidence that a main housing risk factor for foot problems was being kept on hard surfaces [23]. Similarly, more concrete flooring on walking routes in Thailand was associated with higher FS in the univariate analysis. Studies in cattle have shown that animals kept on hard surfaces had an increased incidence of foot lesions and lameness [59,60], whereas those with access to pasture had fewer problems [61]. In zoo settings, the prevalence of chronic foot disease in Indian rhinoceros (*Rhinoceros unicornis*) was 22.2% and was suspected to be linked to concrete flooring and lack of access to ponds and wallows [62].

An unexpected finding in North America was that spending more time indoors appeared to be positively associated with poor foot health, which could be related to how much concrete there is in those spaces [23]. In Thailand, the amount of work and walking distance per day were associated with an increased risk of a high FS in the univariate analysis, possibly because feet and nails are subjected to more pressure when active than when standing still [57]. One factor related to high FSs in both North American and Thailand populations was age [23,29], which was not surprising, and suggests special care should be afforded to geriatric elephants with respect to husbandry and housing. It also is important to note that many foot and joint problems are likely due to the accumulated effects of poor husbandry over time. As such, many facilities have replaced concrete with sand, rubber, or other soft substrates in both indoor and outdoor exhibits, so foot and joint problems are expected to diminish in the future. 

#### 3.2.2. Skin Lesions and Wounds

The prevalence of skin lesions increased with age in both North American and Thailand populations, which probably represents cumulative effects over time. In North America, based on 12 months of medical records, pathologies such as calluses, dermatitis, hyperkeratosis, folliculitis, ulcers, pustules, abscesses, irritations, pruritus, and pressure sores were observed in 37% of the elephants [24]. Skin lesions were distinct from “wounds,” which were inflicted by conspecifics or objects in the environment, and were observed in 6.9% of the elephants. In the survey of Mikota et al. [54], lesions and wounds also accounted for nearly a third of clinical cases. Elephants provided more choice to be indoors or out presented with higher wound scores, which was not expected but could be because free choice is often provided under more extreme conditions; i.e., hotter or cooler climates, or at the peak of summer or winter when skin pathology may be more prevalent [24]. Wounds other than those of the skin were not modeled against management factors in North America, but those identified in Edwards et al. [24] suggest care must be taken to ensure compatibility of herd mates and remove objects that could cause injury.

In the study of Bansiddhi et al. [29], wounds were found in the head region; i.e., forehead and temporal area next to the forehead, which is primarily where the ankus (i.e., hook, bullhook, guide) is used. Sixty percent of the elephants in the study were controlled by an ankus, and of those, 27% exhibited wounds in those areas. Types of ankus wounds included abrasions (80%), lacerations (12%), ulcers (4%), abscesses (3%), and multiple types (1%). Other wounds were associated with the ears (11%), forehead (9%), legs (1%), tail (1%), and other areas (4%). About 40% of the elephants were used in a saddle riding program, although only 5% presented with ulcers in areas in contact with saddle pads or chest pieces. In an earlier study, skin lesions (wounds and abscesses) were found in 265 of 1368 cases (19.1%) throughout Thailand between 2005–2008 and were considered a significant health problem [56]. These rates were considerably lower than the prevalence of 64% reported for 194 elephants at 18 camps in 2007, which showed use of a rice sack as padding was a significant risk factor for having an active lesion [63]. Camps in northern Thailand, where the study [29] took place, generally use hammered bark, blankets, or sponge material as saddle padding [4], which may have lowered the incidence of saddle injuries. Resting on sand floors at night reduced the risk of a high WS in that study [29], although few facilities provide it in Thailand [4]. Wounds were more prevalent in males than females and related mostly to ankus use, possibly because they have the potential to be more aggressive and need more intensive control. In addition, fights between males can result in serious wounds, including punctures from tusks. 

### 3.3. Fecal Glucocorticoid Metabolites

As summarized in Edwards et al. [64], the primary role of GCs is energy regulation and mobilization [65,66], but at higher concentrations, they facilitate physiological changes associated with the stress response [65]. Stimuli both favorable and unfavorable to welfare can increase GC release; however, most studies of captive wildlife focus on how prolonged exposure to psychological or physical stressors increase GCs and negatively affect well-being, such as causing immunosuppression, decreased wound healing, increased susceptibility to disease, poor reproduction, and development of stereotypic behaviors [67]. The use of FGM analyses to monitor adrenal cortical activity has been demonstrated in elephants under a variety of normal physiological (e.g., reproductive states, seasonality) and stressful (e.g., logging, translocation, inadequate environments) conditions [17,30]. In North America, factors associated with higher FGM included being housed alone [17], which also was a risk factor for stereotypic behavior [15]. Lower concentrations were associated with more exercise and enrichment, and a variety of space variables such that having the choice to be in or out may modulate adrenal activity by affecting an animal’s perceived control over its environment [68], which agrees with observations by Greco et al. [15] that those same factors were associated with reduced frequency of nighttime stereotypy.

In Thailand, exercise in the form of riding was associated with better BCSs, lower FGM concentrations, and healthier lipid and metabolic profiles [26,27,28]. In fact, elephants at one of the observation-only camps had the highest FGM concentrations, were fatter, and had poorer metabolic function [27,28]. Those elephants were in close proximity with tourists and mainly stood in one location to be fed treats, with limited time for walking or socializing. In one study, higher FGM concentrations were observed during the high tourist season compared to the low tourist season in Thailand and were related to more restrictive confinement, work type (with no riding activities > riding), and less exercise and so may have been related more to higher tourist numbers than work intensity. Positive relationships of FGM to dietary quantities likely were connected to obesity (i.e., high BCS). Studies in many species have touted the benefits of exercise to physical, physiological, and psychological health, but only recently have studies shown it to improve biological function in elephants. Thus, claims that tourist activities involving riding are universally bad for elephants is too simplistic. However, we need more data on primarily observation-only camps to determine how limited activities affect adrenal activity and health. 

Elephants had lower FGM when kept in open areas at rest (e.g., they were able to move freely and socialize) and when chained in the forest at night (move more freely in a less artificial environment) [30]. That study also found that elephants with major wounds (WS = 2) had higher FGM than elephants with no visible wounds (WS = 0), which was not surprising because ankus use was associated with higher WS in the larger study [30]. By contrast, in North America, elephants at zoos where the guide was used actually had lower FGM, perhaps because it was clearer to the animals what was being asked. Thus, in and of itself, the ankus does not directly contribute to poor welfare; only its misuse does. In Thailand, two of the authors (C.T. and P.B.) have observed in some places where the ankus is prohibited that mahouts may resort to other means of control, like hiding nails or knives, or having strategically placed ankuses. There are no data on how prevalent this is, but in our view, it highlights a problem when some handlers feel the need to hide essential tools to appease animal activists, even if there was no abuse, which can create an unsafe environment for elephants and handlers working in free contact.

In zoos, elephants with joint problems had higher FGM [17], which could be related to increased pain [69] and to spending more time on hard surfaces and being older [23]. A similar relationship between FS and FGM was not found in Thailand; that may be because use of concrete already is more limited [4], and we anecdotally noted that cases of arthritis and other joint problems are comparatively rare. Compared to females, bulls in tourist camps exhibited higher FGM concentrations in association with higher WS, which again may be because they often require more control through ankus use or chaining [4,30]. Bulls present a particular management challenge in both regions and are often isolated (zoos) or kept on short chains (Thailand) for prolonged periods of time, especially during musth, as a safety precaution. Thus, appropriate steps must be taken to ensure the welfare needs of bulls are met without compromising human and animal safety—a significant challenge with no clear solutions as of yet.

Mean concentrations of FGM in elephants in Thailand were less than half those of elephants in North America, although there was overlap in mean concentration ranges: 11.4–194.7 ng/g for Thai compared to 59.7–282.9 ng/g for North American elephants. Some of these differences may be due to slightly different extraction methodologies. Although the same antibody and EIA protocol were employed, the fecal processing technique differed (conventional oven drying in Thailand versus lyophilization in North America; sifting of dried feces to obtain a purer sample with less fiber; multi-tube vortexer in North America versus boiling extraction in Thailand), which could result in differences in steroid extraction efficiencies. Thus, we recognize the need to further explore regional differences using more standardized techniques.

### 3.4. Stereotypic Behavior

In North America, we found nearly a quarter of Asian elephants performed stereotypic behaviors during the day and over a third at night, the majority of which involved repetitive motor movements [15]. Specific factors associated with stereotypic behaviors were evaluated in North America only and related to a variety of demographic, social, husbandry, and management variables. Males stereotyped more than females, as did those involved in more zoo transfers and exposed to different feeding methods. Stereotypies also were more common in association with using positive rewards, perhaps related to anticipation of receiving a treat for good behavior. Several space experience variables and being exposed to a herdmate death were also associated with higher rates of stereotypy [15]. By contrast, being exposed to multiple elephants or separate social groups, increased enrichment and feedings, more choice, and housing with mixed sex and groups with juveniles were related to lower stereotypic behaviors [15]. As with previous studies [70,71,72], our findings show that a good social environment is crucial for the psychological health of elephants, at least based on using stereotypic behavior and FGM as welfare outcomes. Associations with the time elephants were directly managed could be related to sociality as well, in that positive elephant-keeper relationships may buffer stress, thereby protecting against stereotypic behavior risk. In fact, Carlstead, et al. [21] found for Asian elephants that low serum cortisol was associated with positive interactions of keepers with elephants, positive behaviors of elephants (friendly, affiliative), and elephants that interacted with the public. Transferring elephants between zoos also can have major social implications due to separation from herdmates and caretakers, and introduction to unfamiliar elephants and humans. Social instability can elicit a variety of stress responses and has been linked to stereotypic behavior performance in elephants [71] and other species [73]. While there are any number of potential interactive pathways that contribute to elephant stereotypy, the connection to sociality is particularly compelling given the highly social nature of elephants [15].

The only housing variable associated with stereotypies in North America was percent in/out choice, which presented a reduced risk. That may be attributed to the increased complexity of a “mixed” environment that affords elephants the opportunity to choose between environmental options [74]. Two other space variables that weighted the size of various environments by the amount of time elephants spent in them throughout the day and night also were associated with reduced stereotypy risk. This expectation was based on findings from other studies showing that stereotypic behavior in elephants is closely linked to the amount of space they have access to [75,76,77]. While amount of space per se was not included in the models, it appears to be the quality and not necessarily the quantity of space that is most important to good welfare [15], and this would be no less true for elephants in Asia. It should be noted, however, that Greco, et al. [15] modeled Asian and African elephants together even though there were significant species differences in rates of stereotypies (Asian > African). It is very likely that risk factors would be species-specific, so additional studies are needed that take that into consideration. 

In Thailand, a quarter of elephants also exhibited stereotypic behaviors based on mahout interviews. This was lower than expected, so we suspect it may be underestimated due to lack of mahout knowledge of what stereotypic behavior is or what it means and to limited observation times. It also is possible that elephants may not stereotype as much when mahouts are present, and we know from personal observation (C.T., P.B., J.L.B.) that elephants will stereotype when chained. It is important to point out that the stereotypic behaviors may reflect past environments that were suboptimal, and not current conditions [12,78], although regardless, they indicate welfare is not optimal. Lower FGM were observed in elephants that exhibited stereotypic behaviors, which may represent a means of coping with a stressful environment [78,79]. Based on neuropsychological tests and measures of FGM concentrations in captive rhesus macaques, stereotypies were found to be related to brain pathology, and an adaptive mechanism to better cope with stress [80]. While lower FGM concentrations in tourist elephants that stereotyped suggests it may be a coping mechanism, there is little argument that these behaviors develop under suboptimal conditions and should be mitigated by improved management. A comprehensive behavioral survey of elephants in Thailand is therefore warranted, particularly because risk factors for display of stereotypic behaviors identified in other studies, such as chaining, boredom, social isolation, and frustration [12,15,81] are concerns for Thailand tourist elephants [4].

## 4. Conclusions and Future Directions

Here, we review our collective work to define metrics and identify risk factors that can inform on population and individual-level elephant welfare needs and solutions. Although epidemiological information is by nature correlational only, the results provide a starting point for additional studies based on aspects of husbandry and management found to be associated with welfare outcomes in North America and Thailand. As our two datasets show, a number of factors important to elephant welfare are comparable between regions, despite how divergent the specific management practices is. It is clear that elephants do better in more naturalistic environments that are enriching and meet social needs, while it is important that diets and exercise be balanced to ensure healthy body condition and metabolic function. Foot health was similar between elephants in North America and Thailand; few had serious problems, and most were related to nail cracks. It was clear from both studies, that keeping elephants on soft substrates is key to good foot health. Types of wounds did differ, however, with problems being related more to husbandry and skin care in North America versus improper use of equipment (e.g., ankus, saddles) in Thailand. The AZA announced it will phase out ankus use by the start of 2021, and today, most elephants are now managed in protected contact. However, carrying an ankus is often necessary for the safety of elephants, mahouts and tourists in free-contact situations, which is how elephants are managed in Thailand. Our study only evaluated a small percentage of the tourist elephant population and found about a quarter of elephants had wounds associated with ankus wounds and 5% with saddle equipment. While the vast majority of elephants showed no signs of abuse, protocols that prevent the misuse of equipment and unnecessary wounding should be created and then enforced. The science does not support claims that riding elephants per se is harmful, although saddles and padding must fit properly, and work hours controlled. Studies do show, however, that feeding high calorie items may be harmful to physical and physiological health, so if elephants are not used for riding or encouraged to exercise, treats should be limited.

Results are now being applied across venues. In North America, zoos are modifying exhibits to diversify feeding, promote exploration and exercise, and create compatible social groups. In Thailand, many camps now limit how much tourists can feed elephants, with some replacing high-calorie treats with natural forage. A science-based set of welfare guidelines is being developed in Thailand to encourage more freedom for elephants to engage with their environment and conspecifics and rely on more positive training and control techniques. Thai veterinarians and researchers are working with tourist agencies and governmental organizations to encourage regulations that will promote maintenance of healthy, sustainable populations and inform tourists of ethical venues. Moving forward, we envision future studies will build on these results, identifying additional measures of welfare, and integrating experimental components to further elucidate the complex connections in the daily lives of elephants and how they affect physical and psychological states. In addition, it will be important to study the effects of early life experiences such as birth origin, rearing history, training methods, physical and social environments, facility transfers, and work activities among others on long-term health and welfare outcomes [82]. For example, in free-ranging African elephants, both maternal and environmental factors in early life have long-lasting effects on offspring [83]. Specifically, maternal inexperience, slow growth during early lactation, and exposure to drought in early life was associated with reduced growth rates of calves, smaller adult size, delayed first reproduction, reduced lifetime survival and ultimately limited reproductive output [83]. In Myanmar timber elephants, female calves born in “stressful” months when maternal FGM concentrations were higher in association with heavy workloads and monsoon conditions, exhibited faster reproductive senescence in adulthood and had an overall reduced lifetime reproductive success than cohorts born at other times of year [84]. In that same population, it was further shown that while early-life reproduction impaired female survival, overall lifetime fitness was higher [85]. Thus, a greater investment in reproductive success early in life is evolutionarily favored by higher lifetime fitness despite costs to later-life survival. Physiologically, African elephants giving birth even once had a decreased risk of developing ovarian acyclicity and abnormal prolactin secretion [86]. Lifetime experiences can also affect health, many of which are due to a lack of attention or knowledge of how diets, exercise and substrates impact individual elephants. The relationship between foot problems and hard surfaces shown in our US and Thailand studies is a good example [23,29], with evidence suggesting that facility and management changes to replace concrete with dirt or sand ultimately will lead to future improvements in foot and musculoskeletal health, and therefore, welfare [23]. Finally, behavior can be impacted by prior experience as demonstrated by the US study that showed a higher risk of stereotypy in elephants that experienced more inter-zoo transfers [15].

In conclusion, concerns about animal welfare increasingly shape people’s perspectives on the acceptability of having animals managed under human care. We contend that while there is no “one-size-fits-all” management strategy that can guarantee good elephant welfare across all venues, there clearly are right and wrong ways to manage them. Epidemiological and empirical studies are important for establishing evidence-based husbandry and management protocols that meet individual’s physical, physiological, and psychological needs, and helping the public make informed decisions on the ethics of wildlife venues. Though conditions vary between zoos and tourist camps, there are commonalities in what elephants need and how management can be improved to support good welfare.

## Figures and Tables

**Table 1:** Description of independent variables for General Estimating Equation models of welfare outcomes in epidemiological studies of Asian elephants in North America and Thailand.

**Variables—US**	**Definition**
Alternate Feeding Methods	The proportion of all feedings where food was presented in a foraging device, hidden, or hung above the exhibit
Animal Contact	Total count of other elephants with which the elephant shared space. Does not take into consideration how much time was spent with each
Daily Walk Distance	Mean outdoor daily walking distance (km) measured by GPS data logger anklets
Enrichment Diversity	Shannon diversity index score of enrichment activity types and frequencies
Exercise Diversity	Shannon diversity index score of types and frequencies of exercise engaged by elephants
Experienced Herdmate Death	Exposed to the death of a herdmate
Feed Diversity	Shannon diversity index score of feeding types and frequencies conducted by a zoo
Feed Predictability (ref: unpredictable)	The predictability of feeding Times; categorical where 1 is predictable, 2 is semi-predictable, and 3 is unpredictable
Number of Feedings at Night	The total number of times food is provided in at night
Percent Time on Hard Substrates	Sum of monthly percent time spent in environment with 100% concrete or stone aggregate substrate
Percent Time Housed Separately	Sum of monthly percent time spent housed in a social group of one
Percent Time In/Out Choice	Sum of monthly percent time spent in environments where there is a choice of indoors or outdoors
Percent Time Managed	Sum of percent time spent in activities managed by caretaking staff
Percent Time Indoors	Average time spent indoors across 12 months during the day and night housing periods
Percent Time Mixed Sex Group	Average percent time spent in social groups that included at least one member of the opposite sex all months for both day and night housing
Percent Time Outdoors	Average time spent outdoors across 12 months during the day and night housing periods
Percent Time Single Age Group	Average time spent with same age cohorts across 12 months during the day and night housing periods
Percent Time Single Sex Group	Average time spent with same sex cohorts across 12 months during the day and night housing periods
Percent Time with Juveniles	Sum of monthly percent time spent in social groups where an elephant seven years old or younger was present
Positive Reinforcement Training	The relative frequency with which an elephant experienced Positive Reinforcement and/or Negative Punishment over all types of reinforcement and punishment, including treats
Relative Space Experience Change	(Total Day Space Experience—Total Night Space Experience)/(Total DaySpace Experience)
Social Group Contact	Maximum number of unique social groups focal animal is part of
Space Experience In/Out Choice	The average weighted (by percent time) size of all environments in which an elephant spent Time where there is a choice of indoors or outdoors
Space Experience In/Out Choice Day	The average weighted (by percent time) size of all environments in which an elephant spent time where there is a choice of indoors or outdoors during the day
Space Experience Indoors	Average of Outdoor Space Experience across 12 months during both the day and night housing periods
Space Experience Indoors Night	The average weighted (by percent time) size of all environments in which an elephant spent time in outdoor environments only
Space Experience Night	The average weighted (by percent time) size of all environments in which an elephant spent time at night
Space Experience Outdoors Night	The average weighted (by percent time) size of all environments in which an elephant spent time in outdoor environments only
Space Experience Total	Overall refers to an average of measurements across 12 months during both the day and the night
Space Experience Outdoors	Average of Indoor Space Experience across 12 months during both the day and night housing periods
Training Frequency	Derived from Management time budget. Sum of percent time the elephant spent in training, husbandry, play, relationship building, and exercise sessions
Transfers	Total number of inter-zoo transfers an elephant has experienced
Use of Guide	Keeper used a guide when working with elephants
Walk per Week	Number of hours spent with staff-directed walking of elephants each week, ranging from category 1 (< 1 h per week) to category 7 (14 or more hours per week)
**Variables—Thailand**	**Definition**
Ability to Forage	Ability to forage in the forest or on a grassy field every day: yes or no
Access to Water Night	Elephant was offered or had ad libitum access to a water source at night
Primary Diet per Day	Amount of primary diet (kg) consisting of roughage (forage, i.e., bana grass, napier grass, etc)
Supplement per Day	Amount of supplemental foods (kg) generally offered by tourists as treats (e.g., bananas, sugar cane, etc)
Chain Hours	Duration of chaining per day (hours)
Duration Work per Day	Average number of hours elephants worked per day (e.g., giving rides, shows)
Number of Tourists	Monthly estimates of tourist numbers elephants were exposed to at tourist camps
Rest Area Day	Rest areas during the day: shed, open area, tree
Rest Area Night	Rest areas at night: shed, open area, enclosure, forest, tree
Tourist Season (ref: low)	High season (November–February); low season (March–October)
Type of Floor During Work	Type of floor during working hours; dirt only or mix of dirt and concrete
Type of Flooring at Night	Type of floor in rest area during nighttime; dirt, concrete or sand
Use of Hook (ref: yes)	Using a hook to control an elephant; yes or no
Walk Distance Day	Walking distance during working period per day (m)
Walk Time Day	Walking time during working period per day (min)
Work Type	Type of Work: observation, riding with a saddle, riding bareback, no riding, or show

**Table 2:** Mean (±SEM) values for physical and physiological measures in Asian elephants involved in epidemiological welfare studies in North America and Thailand.

Body Condition Score	US			Thailand	
Body condition score [19,20,26,27,28,29]	**Male**		**Female**	**Male**	**Female**
	3.74 ± 0.18 ^a,†^		4.13 ± 0.11 ^b,¶^	2.80 ± 0.04 ^a,¶^	3.50 ± 0.02 ^b,¶^
	Median = 4 (range, 1–5) 2% BCS = 1 6% BCS = 2 19% BCS = 3 31% BCS = 4 42% BCS = 5			Median = 4 (range, 2–5) 0% BCS = 1 5% BCS = 2 26% BCS = 3 40% BCS = 4 29% BCS = 5	
Metabolic status Total cholesterol (mg/dl) [25,26,27,28] Triglycerides (mg/dl) [19,20,25,26,27,28] Glucose (mg/dl) [19,25,26,27,28] Insulin (ng/mL) [19,25,26,27,28] G:I [19,25,26,27,28]	**Male**		**Female**	**Male**	**Female**
	38.21 ± 1.04 ^†^ 12.74 ± 0.59 ^a,†^ 97.00 ± 2.45 ^a,†^ 0.62 ± 0.50 ^a,†^ 253.00 ± 2.24 ^a,¶^		ND 31.63 ± 1.02 ^b,¶^ 101.00 ± 3.14 ^a,†^ 1.56 ± 1.20 ^b,†^ 110.00 ± 9.71 ^b,§^	39.90 ± 0.54 ^a,†^ 26.20 ± 1.11 ^a,¶^ 86.10 ± 1.27 ^a,†^ 0.50 ± 0.05 ^a,†^ 681.00 ± 34.50 ^a,†^	37.40 ± 0.32 ^a,†^ 28.60 ± 0.64 ^a,¶^ 88.90 ± 0.75 ^a,†^ 0.75 ± 0.03 ^b,†^ 196.00 ± 6.72 ^b,¶^
Foot health [23,29]	Median = 2 (range, 0–7) (maximum score = 12) 22% FS = 0 16% FS = 1 16% FS = 2 12% FS = 3 22% FS = 4 6% FS = 5 0% FS = 6 6% FS = 7 0% FS = 8–12			Median = 1 (range, 0–3) (maximum score = 3) 36% at a FS = 0 44% at a FS = 1 18% at a FS = 2 2% at a FS = 3	
Skin lesions and wounds [24,29]	Skin lesions (yes) = 37% Wounds (yes) = 6.9%			Median = 0 (range, 0–2) 77% WS = 0 17% WS = 1 6% WS = 2	
Fecal glucocorticoid metabolites (ng/g) [17,30]	121.55 ± 8.69 ^a^		125.47 ± 4.87 ^a^	56.51 ± 2.42 ^a^	47.74 ± 1.87 ^a^
	124.41 ± 4.90 ^†^			53.49 ± 0.68 ^¶^	
Stereotypic behaviors [15,30]	21% (daytime)	35% (nighttime)		25% (yes), 75% (no)	

G:I = glucose to insulin ratio; FS = foot score; WS = wound score; ^a,b^ Values are significantly different within each region (US or Thailand) (*p* < 0.05); ^†,¶,§^ Values are significantly different between regions (US and Thailand) (*p* < 0.05).

**Table 3:** Comparison of demographic, management, and husbandry factors (independent variables), with positive (+) and negative (−) associations to welfare indicators in Asian elephants from epidemiological studies conducted in North America and Thailand. Includes factors significant in univariate (*p* < 0.15) and multivariable (*p* < 0.05) analyses.

Welfare Indicator	US	Thailand
Body condition score [19,20,26,27,28,29]	Sex (female > male) Age (+) Feed Diversity (+) Positive Reward/Stimuli (+) Feed Predictability (ref: unpredictable) (−) Walk per Week (−)	Sex (female > male) Work Type (observation > riding; riding bareback > riding with saddle) Ability to Forage (+) Access to Water Night (+) Primary Diet per Day (+) Supplements per Day (+) Number of Tourists (+) Chain Hours (−) Number of Feedings of Roughage Day and Night (−) Number of Feedings of Roughage Night (−)
Total cholesterol (mg/dL) [25,26,27,28]	ND	Tourist Season (high > low) Work Type (observation > no riding > riding bareback > riding with saddle > show) Duration Work per Day (+) Walk Distance per Day (+) Age (−)
Triglycerides (mg/dL) [26,28]	ND	Work Type (riding bareback > riding with saddle) Duration Work per Day (+) Number of Tourists (+) Age (−)
Glucose (mg/dL) [19,25,26,27,28]	ND	Tourist Season (high > low) Work Type (observation > riding; riding bareback > riding with saddle) Supplements per Day (+) Age (−) Duration Work per Day (−) Number of Tourists (−) Walk Distance per Day (−) Walk Time per Day (−)
Insulin (ng/mL) [19,25,26,27,28]	ND	Tourist Season (high > low) Work Type (observation > riding; riding bareback > riding with saddle) Primary Diet per Day (+) Supplements per Day (+) Number of Tourists (+) Age (−) Duration Work per Day (−) Walk Distance per Day (−) Walk Time per Day (−)
G:I [19,25,26,27,28]	Sex (male > female) Space Experience Total (+) Training Frequency (+) Age (−) Feed Diversity (−) Feed Predictability (ref: unpredictable) (−) Percent Time Indoors (−) Walk per Week (−)	Tourist Season (low > high) Work Type (observation > no riding > riding bareback > riding with saddle > show) Age (+) Duration Work per Day (+)
Foot health [23,29]	Exercise Diversity (+) Walk per Week (+) Space Experience Night (−) Space Experience In/Out Choice Day (+) Space Experience Total Night (+) Percent Time on Hard Substrates (+) Enrichment Diversity (−) Space Experience Indoors Night (−)	Type of Floor During Work (concrete and ground > ground) Age (+) Duration Work per Day (+) Walk Distance per Day (+)
Skin lesions and wounds [24,29]	Age (+) Space Experience In/Out Choice (+)	Sex (male > female) Type of Flooring at Night (ground > sand) Age (+) Use of Hook (+)
Fecal glucocorticoid metabolites (ng/g) [17,18,26,28,30]	Hours Recumbent Day (+) Percent Time Housed Separately (+) Daily Walk Distance (−) Enrichment Diversity (−) Percent Time Managed (−) Space Experience In/Out Choice (−) Space Experience Night (−) Space Experience Outdoors Night (−) Walk per Week (−)	Rest Area Day (tree > shed > open area) Rest Area Night (open area > shed > enclosure > tree > forest) Sex (male > female) Tourist Season (high > low) Work Type (observation > no riding > riding bareback > riding with saddle > show; riding bareback > riding with saddle) Age (−) Primary Diet per Day (+) Supplements per Day (+) Walk Time per Day (−) Duration Work per Day (−) Stereotypic Behavior (−) Walk Distance per Day (−)
Stereotypic behaviour [15,30]	Sex (male > female) Alternate Feeding Methods (+) Experienced Herdmate Death (+) Percent Time Housed Separately (+) Positive Rewards/Stimuli (+) Relative Space Experienced Change (+) Space Experienced Indoors (+) Transfers (+) Animal Contact (−) Enrichment Diversity (−) Number of Feedings at Night (−) Percent Time Managed (−) Percent Time In/Out Choice (−) Percent Time Indoors (−) Percent Time Mixed Sex Group (−) Percent Time Outdoors (−) Percent Time Single Age Group (−) Percent Time Single Sex Group (−) Percent Time with Juveniles (−) Social Experience (−) Social Group Contact (−) Space Experience (−) Space Experienced Outdoors (−) Use of Guide (−)	Fecal Glucocorticoid Metabolite Concentrations (−)

ND – no data.
